# ‘I don’t know if we can really, really change that’: a qualitative exploration of public perception towards antibiotic resistance in France

**DOI:** 10.1093/jacamr/dlaa073

**Published:** 2020-10-03

**Authors:** Anaïs Essilini, Joëlle Kivits, Frédéric Caron, Jean-Marc Boivin, Nathalie Thilly, Céline Pulcini

**Affiliations:** 1 Université de Lorraine, APEMAC, équipe MICS, F-54000 Nancy, France; 2 Université de Lorraine, CHRU-Nancy, Département Méthodologie, Promotion, Investigation, Nancy, France; 3 Université de Lorraine, CHRU-Nancy, Département des Maladies Infectieuses, Nancy, France

## Abstract

**Background:**

Since the 2000s, French authorities have put in place various national plans to make the general public aware of antibiotic stewardship. Twenty years later, France is still one of the countries with the highest use of antibiotics in Europe.

**Objectives:**

Our study explored the general public’s perceptions of antibiotic resistance, their behaviour around antibiotic use and their expectations regarding awareness campaigns.

**Methods:**

A qualitative study was performed from March 2018 to March 2019 in a French region using focus groups. Two types of public were targeted: parents of young children and retired people. The interview guide contained open-ended questions organized around three main themes: perceptions of antibiotic resistance; experience and use of antibiotics; and health information and campaigns.

**Results:**

Nine focus groups were created, including 17 parents and 19 retirees. Participants did not link antibiotic overuse and antibiotic resistance. Antibiotic resistance was not perceived as a personal responsibility but as a suffered phenomenon on which the participants could not act. The blame was particularly put on the presence of antibiotics in the environment. Although participants expressed trust in their GPs, antibiotics remained perceived as the only solution for them to be cured quickly.

**Conclusions:**

The study highlighted that the GPs were the preferred information source regarding the use of antibiotics. Actions targeting the public and health professionals will have little impact if, at the same time, efforts on work environment representation are not undertaken.

## Introduction

Antimicrobial resistance, and in particular antibiotic resistance, is a global threat. In France, the health authorities have implemented national action plans for 20 years, all including interventions to raise awareness among the general public.^1–5^ Repeated every winter from 2002 to 2007, the nationwide campaign *‘Les antibiotiques, c’est pas automatique’* (antibiotics are not automatic) had an impact on the total antibiotic use (decrease by 26.5%).^6^ From 2009 to 2018, the number of prescriptions per 1000 inhabitants per day continued to decrease (2.81 in 2009 versus 2.38 in 2018), but more slowly.^7^ This decrease was observed in all age groups up to 64 years of age.^7^ Despite this, antibiotic overuse is still an issue in France, which has one of the highest total antibiotic uses in Europe.^8^

To target and evaluate antibiotic stewardship actions, various studies have been conducted to explore general public perceptions of antibiotic resistance.^9–18^ Those studies showed that people behaved inappropriately when taking antibiotics, including poor compliance with treatment or self-medication; knowledge about antibiotics was often poor. The 2018 Eurobarometer survey highlighted that, in France, ‘35% of patients thought that antibiotics are effective against viruses, 16% thought that antibiotics should be stopped when they feel better and only 45% remembered getting information about how unnecessary use of antibiotics leads to their ineffectiveness during the last year’.^18^ This European survey also highlighted that 57% of Europeans and 35% of French people did not know that antibiotics are ineffective against viral infections. For France, this represents a decrease of 6% compared with the survey conducted in 2016.^18^ A survey performed in a middle-sized French city (Nancy, Northeastern France) in 2017 showed that public knowledge was good, with 75.5% and 52.5% of participants, respectively, knowing that antibiotic prescription was not justified for colds and viral infections. The main cause of antibiotic resistance identified was overuse (92%).^19^ Recent studies conducted in the UK, the USA and Sweden also revealed a lack of knowledge about antibiotics and antibiotic resistance, with individuals claiming to know their bodies and thus to know when they need an antibiotic prescription. In addition, if participants identified antibiotic resistance as a threat or an emerging problem they did not consider themselves at risk.^20–23^ All of the recent French studies and most of the recent data in high-income countries are based on quantitative studies^18^^,^^19^^,^^24^ and there are few qualitative studies.^9^^,^^13^^,^^17^^,^^20^^,^^22^^,^^25–29^

While it can be assumed that fighting antibiotic resistance requires the public to be more informed about the risks of antibiotic misuse, ultimately promoting behavioural change, current data tend to show that campaigns targeting the general population are necessary but not sufficient.^30–33^ If the aim is to enhance health campaigns’ potential regarding behaviour change, a better understanding of the general public’s perceptions of antibiotic resistance and what underlies it is necessary. The way in which the individual will understand a health message is a complex social process.^34^ The individual constructs meaning to a received message, drawing on his/her experience and knowledge. For example, cancer screening messages will not be the same for a young person as for a middle-aged person receiving cancer screening invitation letters. In addition, the context in which an individual evolves daily also constitutes a frame of reference in the appropriation of a message.^35^^,^^36^ New strategies to tackle antibiotic resistance aligned with the general public’s experiences need to be devised. It therefore seems essential to better understand the general public’s perceptions of antibiotic resistance and use and what is at the root of these perceptions.

The objective of the present qualitative study was to explore the general public’s perceptions of antibiotic resistance, as well as their attitudes around antibiotic use and their expectations regarding awareness campaigns.

## Methods

To best answer our research objective, a qualitative approach was chosen. We opted for the focus group (FG) technique as it allows collection of a broad variety of ideas, opinions and beliefs on a specific topic.^37^^,^^38^ The study followed the COREQ reporting guidelines.^39^

### Participants and setting

FGs were organized with parents of young children and retired people, two populations consuming more antibiotics than the rest of the general population.^7^

Recruitment modes were diverse. Parents were recruited either through primary schools or sports’ clubs where parents often stayed to watch their children during the training. Retired people were recruited through cultural and recreational groups. The first contact was always an e-mail sent to the institution (schools, sports’ clubs, leisure groups) explaining the study’s objectives. Once the agreement for organizing an FG in the institution had been received, researchers spent time during activities to invite parents/retired people to participate in the FG. FGs were held at the activity’s venues and at one participant’s home for one parent FG. All but one FG were conducted in Nancy, a middle-sized city in Northeastern France, with one FG organized in Paris. All FGs took place between March 2018 and March 2019 and were planned for a 40 to 60 min duration.

### FGs

FGs followed an interview guide constructed around three main themes: perceptions of antibiotic resistance; attitudes around antibiotic use; and expectations towards awareness campaigns. The interview guide was created by F.C. (a public health Master’s student) and reviewed by J.K. (a sociologist), C.P. (an infectious disease specialist) and N.T. (a pharmacist). The guide was tested during the first FG interview and no modification was made. Each FG interview was conducted by two researchers [A.E. (a public health PhD student), J.K. or F.C.] with one leading the discussion and the other one taking notes.

FG interviews were recorded after verbal informed consent was obtained and were transcribed. FG interviews were continued until data saturation was reached. Participation was voluntary and not compensated.

### Analysis

Anonymized transcripts were analysed, once all FG interviews had been performed, using a comprehensive thematic analysis using QSR International’s NVivo 11 software. The analysis grid was constructed by three researchers (A.E., F.C., J.K.); each of the themes and subthemes were discussed within the research team until a consensus on a final list of themes was reached. Each FG was then coded according to the analysis grid.

### Ethics statement

The study was completely anonymous at all stages and no information on the health of participants was collected. No ethical approval was thus required in accordance with French law.

## Results

Thirty-six participants (19 retired people and 17 parents) were recruited while organizing nine FGs (Table 1). Most participants were women (28/36) (Table 1 and Supplementary data). The average duration of a FG interview was 40 min.

**Table 1.** Description of FGs

FG number	Population	Number of participants	Number of women	Mode of recruitment
1	retired people	4	2	computer workshop
2	retired people	2	1	computer workshop
3	parents	4	2	swimming club
4	parents	6	6	swimming club
5	retired people	3	2	leisure workshop
6	parents	2	2	sport club
7	retired people	6	5	association
8	retired people	4	3	association
9	parents	5	5	school

Three main issues emerged from data analysis: knowledge and perceptions of antibiotic resistance; the ambiguous approach to antibiotic prescription; and the social role of antibiotics.

Illustrative verbatim quotations are listed in Table 2.

**Table 2.** Selection of the most illustrative verbatim quotations

FG	Verbatim quotation
What is antibiotic resistance?	
FG6	‘For my part, no, I didn’t try to find out’
FG1	‘It’s a scary subject, I find it scary’
FG7	‘I am part of a generation where there were not many antibiotics in our youth, so we react well, it’s a matter of habituation’
FG2	‘you certainly have to take it, it’s from a certain amount not [that you become resistant]’
FG9	‘no no no no no, it is not transmitted […] each body is different and reacts differently (everyone agrees)’
FG3	‘I don’t know if we can really, really change that’ [antibiotic resistance]
The responsibility of others on an individual burden	
FG1	‘Because the drugs, we can be careful, I can only take what I really need, but we don’t know elsewhere, if the antibiotic resistance wouldn’t also come from that, from those tiny doses that we eat all the time’
FG2	‘to be in contact with antibiotics hidden in food or meat, or whatever, the body no longer defends itself even if the antibiotics are given’
FG3	‘So I don’t know if we, as an antibiotic consumer, can really, really change that […], we feed animals antibiotics all the time, […] will taking a little less antibiotics makes us feel better?’
FG7	‘Farmers have the same approach as they do with their animals. Today, it’s antibiotics at all costs, so […] they measure remaining traces in the food, of pesticides, but also antibiotics […] they used and continue to use far too much, especially on intensive farms.’
My trust, my GP	
FG9	‘I think they were more warned about it; before, the antibiotic was the solution and that’s it’
FG1	‘Awareness campaigns coincides with need to cut public costs’
FG8	‘The Ministry wants to save money, but the working mother has to come back, and the child suffers for three or four days’
FG4	‘No, I trust my doctor’
FG2	‘when I call for help, saying “I came because I really need to be accompanied”, if the treatment is not effective, I go back 3 or 4 days later, in a pitiful state, so we need antibiotics, it seems obvious to me, essential. It’s the doctor who prescribes antibiotics, it’s not me who will ask for it, but it’s obvious that I want my little dose’
FG8	‘when I go to the doctor, it’s because I’m really feeling sick, but I may not express it loud enough, so I’m not given antibiotics, which makes me go several times’
FG1	‘If they say that we must give less (antibiotics) or eliminate them, they must give another solution to replace them, by what we replace them, because if we are told we must not take many, but if we are sick what should we take instead’
FG7	‘we’re not going to be suspicious of antibiotics when they’ve saved so many people, I don’t agree, if there weren’t antibiotics we wouldn’t be here’
The unspoken consequences of being sick	
FG5	‘Only antibiotics cure you […], it takes much longer with anything else’
FG2	‘there are times when I resisted to get them but I really needed to get back to work’
FG7	‘going back to work and putting the children back to school […] antibiotics allow that […] fastest solution’
FG3	‘With no prescription you don’t feel well, you feel less healed than when you have a prescription’
FG8	‘eight days twice a day and well, there are still 3/4 pills left. I keep those 3/4 pills and add them to previous ones, that makes me 8 pills so it serves me the week I still have a problem’

### Knowledge and perception of antibiotic resistance

#### 1. What is antibiotic resistance?

When asked about antibiotic resistance, most participants had never heard of it before. They had difficulty explaining the term and often resorted to their experience or one of a relative. In most FGs, the consequences of antibiotic resistance were not a concern and remained unclear and distant. The awareness obtained during the FG interviews about the severity of the situation was alarming for some participants. They were confused about the mechanism of antibiotic resistance, often described as the body becoming resistant. According to participants, overuse of antibiotics would lead to ‘habituation’: the bodies of individuals who took too many antibiotics would no longer respond to treatment. Another cause of ‘habituation’ could be the failure to comply with the prescribed treatment duration, which would allow the disease to return more strongly. Participants had an individual and bodily vision of resistance. They were positive one can build immunity, but antibiotic resistance cannot be transmitted from one person to another.

#### 2. A risk for the individual generated by others’ responsibility

When they tried to explain the main causes of antibiotic resistance, participants mentioned the presence of antibiotics in the environment, particularly linked to agricultural activities and water. The blame was put on farmers who irresponsibly give antibiotics to their animals and on people who did not properly dispose of their antibiotics, with these ending up in soil or water. Transmission between humans and the environment was perceived only in the direction of the environment to humans and was worrying.

Antibiotic resistance was not felt as a personal responsibility but as a suffered phenomenon on which the participants could not act. The 2002 awareness campaign ‘antibiotics are not automatic’ was well accepted by all generations and mentioned by almost all FG participants. They perceived the message, however, as emphasizing the GP’s sole responsibility.

### The ambiguous approach to antibiotic prescription

Regarding their behaviour toward antibiotic prescription, participants made a distinction between the medical community, health authorities and their GP.

Participants were particularly suspicious towards the medical community. Except for their family GP, ‘doctors’ were described as prescribers who did not take the time to come up with a proper diagnosis during the consultation. Participants distinguished the ‘old generation’ from the ‘new generation’. The new generation was described as more informed, prescribing only when necessary and performing diagnostic tests, unlike the ‘old generation’ who used antibiotics because it was quick and easy.

Antibiotic awareness campaigns were seen as an attempt by the health authorities to decrease medication costs and the deficit of the French National Health Insurance. Cost reduction was viewed poorly, especially in a context where antibiotics enjoyed great popularity and were considered as an absolute necessity. Participants negatively saw the perceived priority set by the government on health budget cuts and its failure to consider the consequence of sick leave, which also has a cost. In addition, they were condemning the pressure exerted by health authorities on doctors to reduce their prescriptions.

Despite their expressed mistrust of authorities, health agencies and the medical community, the participants expressed an entire trust in their doctors. They described their family GP as someone who understood them and ‘knew them’. They felt they could discuss the prescription with him/her but would respect their decision no matter what.

### The social role of antibiotics

#### 1. The miracle of antibiotics

Although some individuals admitted overusing antibiotics and requesting them when they were not recommended by their doctors, participants felt they had a good knowledge of their bodies and only took them when necessary. Indeed, in their expectations of being cured, they often felt frustrated if no antibiotics were given to them when they felt really sick. While they considered that they did not ask for antibiotics, many of them returned to see their doctor after 3 or 4 days if they did not feel better. Other participants expressed their surprise that no antibiotics were prescribed to them. The support provided by the doctor to the patient during the healing process was often synonymous with the prescription of an ‘effective treatment’ and antibiotics were frequently regarded as the treatment of choice. Antibiotics were perceived as a special treatment and the only solution for them to be cured quickly.

#### 2. The unspoken consequences of being sick

When asked about the non-prescription of antibiotics, all generations of participants expressed concern about sick leave. Sick leave was negatively perceived and difficult to experience; staying at home to rest for a common infection without any ‘effective’ treatment was an idea badly accepted by participants. Although they were aware that antibiotics were not effective for viral infections, the need to be active—for parents to be at work, for retired persons to take care of their grandchildren or being able to do their activities—was again their main priority regardless of their understanding of antibiotic use. Some participants, in order to prevent consulting their GP, were prone to self-medicate or medicate their children. Leftovers of previous antibiotic prescriptions were then preserved and sometimes leftovers of different treatments were combined. Antibiotics were seen as the only solution to cure any infection quickly. If they were not prescribed, participants were expecting an alternative treatment.

## Discussion

### Summary of main findings

Participants had partial knowledge about antibiotic resistance and its mechanisms. Its consequences were not experienced as a concern or seen as a close threat and the predictions were considered alarming. Antibiotic resistance was not perceived as a personal responsibility.

In their perception of antibiotic resistance stakeholders, they distinguished health authorities—whom they mistrusted—and the medical community—deemed to have little competence—from their family GP, whom they generally trusted.

The idea of being powerless over their illness and having to wait for a spontaneous recovery was very frustrating and difficult to accept. Despite their knowledge of the ineffectiveness of antibiotics for viral infections, the need to stay active and their negative perception of sick leave led them to set up strategies to get antibiotics. Some participants returned to their doctors several times while others kept leftover antibiotics.

### Comparison with existing literature

Despite numerous awareness campaigns targeting the general population in France, participants had partial knowledge on antibiotic resistance. As described in many studies, participants thought it was their bodies that were becoming resistant, not the bacteria.^17^^,^^20^^,^^25^ Although they did not feel responsible for the increase in antibiotic resistance, participants were very concerned about it. American and British studies have shown that unlike French and Swedish patients, most people in these countries did not identify antibiotic resistance as a threat.^21^^,^^22^^,^^25^ The causes of antibiotic resistance identified by French participants were their presence in the environment and their overuse in agriculture and animal breeding, which has not been reported before to the best of our knowledge.^17^^,^^21^^,^^28^

As is well described in the literature, antibiotics were considered as the miracle treatment and a validation of the severity of the disease.^20^^,^^27^ Despite their understanding of the ineffectiveness of antibiotics for viral infections, the non-prescription of antibiotics was seen as a minimization of the severity of the disease.^20^^,^^25^^,^^27^

Participants made a real distinction between the trust they have in their family GP, the suspicion towards the medical community and the disapproval of economic choices perceived to be driving health authorities. To the best of our knowledge this result had never been observed or reported. This may be explained by crises and health scandals having amplified distrust attitudes towards health expertise in France and in the rest of the world over the last few years.^40^ Confidence in experts’ assessments has become somehow relative and this phenomenon appears generalized in contemporary society.^41^^,^^42^ Regarding antibiotics, the question is not so much the validity of scientific knowledge, often doubted such as in the case of vaccination,^43^ but the faith placed in authorities whose interests are not perceived as being those of patients. Being suspicious of the medical community and authorities corresponds to a reflective positioning and a generalized attitude of mistrust toward institutions, characteristic of risk societies.^41^^,^^44^^,^^45^ In such societies, scientific communication is often challenged.^46^ However, our study shows the centrality of the doctor–patient relationship, particularly within uncertain times.

Often seen as an effective treatment and a way to heal quickly, participants expressed that antibiotics constitute an effective treatment to avoid sick leave. Sick leave was negatively perceived and difficult to experience for participants, therefore leading to overuse of antibiotics. This reflects another societal issue: the relationship between work and health status. While there exists today abundant literature on how patients with chronic disease, disability or long-term illness may encounter difficulties and need to adjust to the work market—and possibly on how the work environment finds solutions to keep them at work—the question of sick leave for acute illnesses, to our knowledge, is poorly documented. However, our study results highlight the social pressure for French people to return to work as soon as possible, and for retired people in our sample to recover quickly to stay active, notably regarding family duties. The phenomenon of presenteeism is today known but often explored with regards to poor workers’ performance and mental health risks;^47^^,^^48^ what remain to be better addressed and understood are social representations of recovery time after acute diseases, such as most viral infections.

### Strengths and limitations

The qualitative approach in this study was particularly appropriate; the FGs, through group dynamics and confrontation of points of view, permitted participants’ poor behaviour to be situated within their perception of antibiotics as miracle cures and antibiotic resistance as a distant threat. Identification and description of these perceptions and misconceptions will help target and implement specific interventions. However, the study presents some limitations. A possible selection bias cannot be excluded because: (i) those who agreed to participate are probably more knowledgeable about antibiotic stewardship than those who refuse; (ii) the sample had an over-representation of women, who are known to have better knowledge of antibiotics than men;^18^ and (iii) retirees and parents of young children were targeted because they are heavy consumers of antibiotics, and therefore probably have knowledge and perception of antibiotics that cannot be generalized to the general population.

### Implications

Widely promoted these days, the notion of ‘One Health’ is becoming increasingly important for antibiotic resistance. The interviews showed that participants were very concerned about the presence of antibiotics in the environment and its consequences for their health. The participants’ interpretation of the presence of antibiotics in the environment (in particular in water), perceived to be mostly due to animal breeding, for example, was for them the main cause of antibiotic resistance. They denied their own involvement and responsibility for their suboptimal behaviour around their use of antibiotics. It is therefore crucial that awareness campaigns include a coherent One Health approach, to avoid misinterpretation and suspicion from the general public. This is all the more important since we are currently living in a context where one builds up a distrust in the authorities.

Participants expressed their doubts and dissatisfaction with political decisions affecting their daily lives. Any development of public health interventions must consider that the population is distrustful from the outset. The current context and its implications on health policy interpretations will not be changeable through public health interventions.

During the interviews, the need to be treated with antibiotics even in the case of viral infections highlighted an important drift of the idea ‘I can’t be sick’. Antibiotics are considered to be the miracle cure. Going home from the doctor, feeling very sick, without any antibiotic because the infection is viral, was unthinkable for the participants. Limiting the treatment to symptomatic medications is perceived as a lack of acknowledgement. Messages in awareness campaigns often associate viral infections with a benign and self-limiting illness. Asking individuals to be patient and to let the disease progress seems very poorly accepted. A spontaneous evolution of the disease towards healing over time, with a positive perception of the self-healing capacity of the body, are counterintuitive ideas as contemporary society leaves little place for disease and inactivity.^49^

Patients’ trust in their family GP has made them the ideal ambassadors for antibiotic stewardship. This privileged position is a double-edged sword. Many high antibiotic prescribers are not convinced they overprescribe. In turn, their patients are unaware of good prescribing practices and current awareness campaign messages are not very effective on them. It is therefore a priority to inform and train doctors, to assist them in changing their communication and prescribing habits, as they have an essential role to play.

### Conclusions

This study showed that antibiotic resistance was considered an emerging problem by French people. Awareness campaigns targeting the public should take into consideration the following key findings: GPs play a vital role in the communication around antibiotic resistance and should be part of a wider One Health approach. Furthermore, actions targeting the public and health professionals will have little impact if, at the same time, efforts on work environment representations are not undertaken.

## Supplementary Material

dlaa073_Supplementary_Data
