# Supplementary material for: ‘I don’t know if we can really, really change that’: a qualitative exploration of public perception towards antibiotic resistance in France
Source: JAC Antimicrob Resist. 2020 Oct 3;2(3):dlaa073. doi: 10.1093/jacamr/dlaa073 (PMC8209967; doi:10.1093/jacamr/dlaa073)
Supplement: dlaa073_Supplementary_Data [file dlaa073_supplementary_data.docx]

**Supplementary data**

**Sociodemographic data survey**

Sex:

Age:

Domicile:

Marital status:

- Married
- Single
- Divorced
- Widowed

Number of minor children:

Level of education:

- None
- Diploma of Higher Education
- A level
- Bachelor
- Master
- PhD

Employment status:

- Unemployed
- Retired
- Student
- Employed

Estimated state of health:

- Very good
- Good
- Poor
- Very poor

Chronic health problem:
